# Case of Extensive Caries of the Fifth and Sixth Ribs, and Disorganization of the Greater Part of the Right Lobe of the Lungs; with a Description of the Operation for the Same, &c.

**Published:** 1824-02

**Authors:** Milton Antony

**Affiliations:** Philadelphia Journal.


Art. VI.—*Case of extensive Caries of the fifth and sixth Ribs, and Disorganization of the greater Part of the right Lobe of the Lungs; with a Description of the Operation for the same, &c.* Communicated by Milton Antony, M.D.—*(Philadelphia Journal.)*

On the 3d of March, 1821, I made my first visit to Elmon Allen, an interesting youth about seventeen years old. The preceding part of the history of his case was substantially as follows:

Between two and three years before, he had, by a fall from his horse, received a severe injury on the right side, whereby the sixth rib was believed to have been fractured. Considerable ecchymosis immediately appeared. As the pain, however, did not long continue severe, no dressing whatever was applied to the wound, and in a few days little attention was paid to the case. The local irritation and ecchymosis measurably, though not entirely, abated; and, during the two years next following, he was frequently able to walk or ride, without serious inconvenience. Several times, however, during this period, he was, in consequence of irritation and tumefaction produced in the injured part by over-exertion or accident, forced to keep his bed for days, and sometimes for weeks. Such, at one period, was the inflammation, that a blister was raised on the part. Thus the case proceeded until about the end of 1820, when the pain in the part became distressing. Soon after this, a pungent and extremely severe pain was fixed at the vertebral and sternal articulations of the injured rib. So great was the local irritation, that a constitutional disturbance was excited, and a fever of hectic type ensued. This continued with severe pains at the extremities of the sixth rib, with a cessation of pain in the wound, which was followed by an unpleasant feeling of weight and distension. About this time it was observed that there was little or no motion in the right side of the thorax in respiration.

On my arrival, I found the patient in good spirits, though considerably emaciated, and had not rested at night for some length of time without laudanum. Respiration was laborious, with much exertion, and dilatation of the nostrils at each inspiration. The motion of the heart was strong and sluggish, and imparted to the touch a sensation somewhat similar to that which it gives when surrounded by water in the pericardium, or where there are adhesions to that membrane. Each pulsation was distinctly perceptible to the eye, in three intercostal spaces on the left side of the thorax; but these pulsations had the appearance of slow, heavy undulations. No more respiratory motion was perceptible in the right side than appeared to be the effect of the repeated inflation of the left lobe, and consequent motion of the parietes of the left side, to which the parietes of the right were attached. The pulse was laborious, more than ordinarily full, and in frequency about one hundred in a minute. The tumefaction of the part was in the direction of the ribs, extending from the sternum to the anterior edge of the latissimus dorsi; about two inches in width, and one in height at the most elevated point, which was several inches immediately over the injured part of the sixth rib. The skin covering the tumor was entire, and without discoloration or change of appearance, except a slight enlargement of superficial vessels.

My friend Dr. Pugsley, an aged, experienced, and accurate surgeon and anatomist, whose obliging assistance I had the satisfaction of obtaining in the case, did not arrive until three o'clock P.M. In consultation, we agreed that it was probable one or more ribs were carious, and that an extensive abscess had formed about this carious bone, the contents of which gave tension and elasticity to the tumor externally, and the inward projection of which made such pressure on the right lobe of the lungs as to impede the function of respiration on the right side, and prevent the free circulation of blood about the bronchial tubes of the right lobe of the lungs, which only takes place during their inflamed state. It was further agreed that an incision should be made in the direction of the ribs, with the motive of removing such parts of them as might be found carious; and then be governed, in the subsequent part of the operation, by circumstances.

Every necessary preparation being made, at four o'clock the patient was placed supine on a suitable table, the right arm being extended by the side of the head, and that and the left arm supported by assistants; I commenced my incision at the sternum, and extended it freely through the super-incumbent integuments, immediately over the space between the fifth and sixth ribs, down to the lower end of the tumor. The fleshy digital origin of the serratus magnus from the sixth rib,—the interlocking fibres of the origin of the obliquus externus abdominis,—the costal origin of the pectoralis major, and the intercostal muscles, with their proper vessels, having, by the resources of the system, been removed prior to the operation, no blood-vessel of particular importance was cut. A copious discharge followed, not of pus, (for not a particle of purulent matter was observed,) but a kind of grume and old red coagula, intermixed with dense coagula of lymph, resembling irregular fragments of membranes. This substance seemed to occupy the whole of the tumor, and surrounded entirely the carious bone. On the escape of that part of it which rested on the anterior portion of the ribs, the fifth and sixth ribs presented to view.

The sixth rib was found of a caseous consistence and brownish colour, from its sternal articulation to the other end of the wound. The anterior half of this rib was easily removed by the fingers in small fragments, leaving the sternum apparently healthy, immediately behind the anterior end of the under lip of the wound; and the end of the vertebral fragment at the posterior end of the wound also apparently healthy, and attached to the surrounding parts. The inferior edge of the fifth rib, opposed to that part of the sixth which I suppose to have been the seat of the first injury, was in the same condition for about two inches in extent. A section of this rib, on being cleared of the surrounding parts each way to the place where healthy adhesion existed, was removed by cutting forceps, these being found competent to the purpose, without so enlarging the incision as to make room for the saw. Here the intercostal artery of the fifth rib was cut, and secured by ligature. Having detached those portions of the fifth and sixth ribs, and my opening through the bony structure of the side being free, I proceeded to remove the grumous and coagulated matter within the ribs. This being accomplished, I observed a difference in the quality of the disorganised substances remaining. It now began to exhibit a more homogeneous appearance, more broken in texture, and of a very dark greyish colour, occasionally tinged with a crimson hue. The depth into the thorax, to which I had now penetrated, being greater than the diameter of the ulcerated part of the parietes of the thorax from above downward, and from the ribs to the integuments, and this added to the different appearance of the disorganised matter, excited my fears for the safety of the lungs. I immediately determined on making examination with my fingers for the lungs, by penetrating as deep into the substance beneath as I could without resistance. This I immediately proceeded to do, and, to my utter astonishment and mortification, I was able to penetrate the right cavity of the thorax, with my first and second fingers, in every direction, to the full depth of three and a half inches, without any resistance more than the pulpy substance before described was calculated to give, except occasionally meeting with more or less disorganised fragments of the bronchial tubes; all of which tended plainly to prove to me that the substance of the right lobe was extensively destroyed. Thus disappointed in my views, and fearing to distress the mind of the patient by making a candid statement of the case to my friend Dr. Pugsley, I directed the finger of the latter into the wound, and he made a full and satisfactory examination to the same effect. I will here observe that, in this examination, no respiratory motion was perceptible to the fingers. One minute's reflection dictated to me the only course which could possibly be pursued, and which I immediately adopted. It was carefully to take away with my fingers all the parts within my reach, which were removeable without violence, or the danger of producing hemorrhage in the event of my reaching near any sound vessel. The result of this was the removal of all the disorganised parenchyma of the lungs within the reach of my fingers, (judged to be between one and two pounds in weight,) carefully cleansing it from between and around several of the branches of the bronchial tubes, which appeared to have retained their texture with more tenacity than their surrounding parts. Having done this, I resigned the completion of this part of the operation to nature, contributing all I could to her assistance in effecting the discharge of the residue,—to what extent I know not. I then cleansed the wound carefully, introducing large pledgets of lint between the cut edges, (which were themselves considerably retracted,) for the purpose of keeping the opening as free as possible for the exit of offensive matter. The dressing was finished by covering the wound with a plaster, and applying a roller so tightly as to prevent the motion of the ribs, causing respiration to be performed by the diaphragm chiefly.

During the operation the patient became weak and faint, and freed from all pain in the part, except the smarting of the cut edges. He rested badly the first night, until he took an anodyne. The first day after the operation he was very weak, breathing laboriously. The room being crowded in the evening, and the air in it rendered impure, he became very much distressed, breathed with increased difficulty, and was thought dying. The room being cleared and ventilated, the patient revived, and rested well that night without an anodyne. The second and third days after the operation, he rested well, with little fever, and slept during the night. The bark was ordered. On the fourth day, when I saw him, he was cheerful, and thought he should recover. The motion of the heart was less laborious. He was quite composed; pulse in the hectic exacerbation 96, natural in force and fullness, though with some febrile quickness and convulsive action. He desired a little weak wine and water, which was given occasionally. By the scent of the wound in dressing he became sickened. The right leg and arm had been a little disposed to cramp since the operation, but was then free from that tendency. He was easily raised by napkins, to apply a many-tailed bandage around the thorax. Dressed with lint, introduced two inches within the ribs, to keep the orifice open; cut surfaces appeared natural and healthy; little suppuration.

Finding the wound had been kept sufficiently open since the operation, and the distance of the patient from me being so great as to render it impossible for me to see him often, or to attend to the dressing myself, I urged the necessity of keeping the wound freely open, by applying as large pledgets of lint as could be introduced. On the ninth day, I received information that he was considerably better; that the discharge from the wound had, since my visit on the fourth, been much more free, and that there was perceptible respiratory motion in the right side. On the eleventh day, I was informed by letter that the symptoms were generally favourable: the wound looked well, mattered freely; slight fever every day; appetite pretty good; has an alvine evacuation daily without medicine.

The wound was closed by granulation in the middle, and open at each end. For a few days previously, it had been washed with decoctum querci; and, on the day before, a hop-poultice was, by the advice of Dr. Pugsley, applied for three hours, and an infusion of hops directed to be used, and continued as a wash. Healthy granulations had been mistaken for fungossities, and burnt alum applied to them.

On the thirteenth day his recovery was, in the minds of himself, his attendants, and friends who saw him, beyond doubt. Tents then penetrated about two inches; but the deepest parts of those cavities which were kept open by them appeared of a flesh colour, and were apparently filling with healthy granulations. He was very cheerful, and had from the wound, the whole of which looked healthy, a discharge of genuine cream-like pus. On the day before he was, by the excessive anxiety of his friends, raised out of bed, and placed erect on the floor. After standing a short space of time, he became vertigenous, and was again put to bed. This movement was performed without any pain whatever, either in the breast or wound.

In a letter to his friends, I again urged the importance of absolute rest. The wounded side of the thorax had increased motion in respiration. It is a fact, not less curious and astonishing than his surviving such extensive lesions of his vital organs, *that no cough at any period of the case, either before or after the operation, more than in ordinary health, occurred.* On the 2d of April, (the thirtieth day after the operation,) he walked about the house, and was desirous of walking over the farm. His father and other friends thought him now out of danger, believing his recovery confirmed.

On the 12th of April, (forty days after the operation,) I was requested to visit him, on account of a little excrescence which had appeared a day or two before, at the under side of the anterior orifice, which orifice was still kept open by a small tent of twisted cotton, about one and a fourth inch long. He was very free from pain, uneasiness, and cough. On examining the wound, I found a soft membranous protrusion, of a livid colour, about an inch in length, and of a conical shape, filling an opening of seven or eight lines in width, which it had made through the granulating edge of the wound at the side of the tent. On the removal of this substance, an opening into the thorax was discovered, through which escaped a substance similar to that which was removed in the latter part of the first operation. Through this opening I was again able to introduce my finger to about the depth of two and a half inches, directly inward, downward, and backward, but not upward. I again removed about half a pound of disorganized substance of the lungs, which had not been able to find its way out before the wound healed. The patient was sensible of my touching in every direction, which was no way more than two and a half inches. The wound was again dressed with as large a tent as the opening would receive, and the old tent above directed to be left out thereafter. The patient remains easy, and has continued to mend to the present time. Respiratory motion is as free in the right as the left side, as far as the fifth rib, below which there is not the least appearance of it.

On the 6th of May, I again visited the patient. His general symptoms were still better. He had at that time so far recovered and improved in health, as to possess all the fullness of habit of his ordinary state. His strength had so far returned as to enable him to exercise on foot freely about the yard, and he felt able and desirous of walking to some place in the neighbourhood, half a mile distant, but was not allowed to exceed the limits of the yard: in short, he was able to sit and walk about the house and yard twelve hours out of twenty-four, with much facility. The wound, in external appearance, remained somewhat as it was at my last visit,—presenting in some places livid projections, and in others growths, not altogether unlike fungus. These livid and fungus-like parts being found insensible and projecting, I suspected some unhealthy substance occupied the cavity beneath. On removing these with scissors and caustic applications, I found a free opening into the thorax, and again removed the contents of the cavity with my fingers. These seemed to consist of coagulated blood entirely, some parts of which were not unlike the buff of blood drawn from patients labouring under inflammatory diseases. I dressed the wound as before, and left the patient.

Notwithstanding the constitutional amendment which Mr. Allen has experienced, in addition to his having survived the loss of considerable portions of two ribs, and not less than two-thirds of the right lobe of his lungs, my prognostication (which in the first of the case was pointedly unfavourable as to its final issue,) is not altered. It is true that I wonder at the powers of nature in continuing the vital motions more than a few hours after the operation, and still more so at the progressive and uniform amendment of the general system: for, in the first instance, I did not believe it consistent with the powers of nature for him to survive the immediate effects of the operation: but my prognostication is now founded on other reasons. There is a large cavity in the right side of the thorax, corresponding in extent with two-thirds of the right lobe of the lungs. This cavity cannot remain an inclosed space, and have its parietes cicatrize: such an idea appears to me not only unprecedented, but incompatible with, and counter to, the grand design in the construction of the human fabric; and, if this be not the fact, it is at least contrary to surgical pathology. Neither can I with reason expect to find the surface of this cavity, together with the edges of the wound, cicatrise, open, and become as a common surface of the body, notwithstanding the lower extremity of the remaining portion of the lungs appears to have adhered or healed in such a manner, that its respiratory function is not in the least affected by the free admission of air into the great cavity below; nor can I believe that the resources of the system are such as to afford any kind of healthy growth, by which this cavity may be filled and remain well. Such a growth must necessarily arise out of the action of vessels which have been constructed for the purpose of regularly furnishing nutriment to the same quantity of parts intended to be reproduced, and their consequent deposition of new substances.

The vessels of the parietes of the thorax, the mediastinum, and diaphragm, have not been designed for this purpose, and are therefore not competent to the task. These are all the parts surrounding, except at the upper boundary of the cavity, where the remaining fragment of the lungs terminates. The structure of the lungs, as well as their function, being entirely unlike that of any other part of the system, I feel, by this peculiarity, justified in the conclusion that their vessels, although they *may heal when injured*, have not the power of *reproducing a lost part.* Hence it is apparent why the cavity cannot be filled with healthy growth, either of lungs or ordinary granulations.

The only other possible ground of recovery, (if indeed there be one,) is the approximation of the sides of the cavity; such as nature would effect in any other part, except the head. This cannot be done, because the arched form of the remaining ribs tends necessarily to keep the cavity from closing by a collapse of its sides. The only curative indication of which I can conceive in the case, therefore, is an unprecedented and desperate resort to surgery, which I should be unwilling to make, and which, most probably, the system would be unable to survive. It is the entire removal of the ribs, from the fifth to the diaphragm, by carefully dissecting them from the muscles and common integuments, leaving these to fall in the cavity, and be applied to its opposite wall for adhesion.

Called in only as consulting physician, and the patient being at a considerable distance from me, I never saw him again. Not long after my last visit to him, I was told that he went out some two or three miles to church; and soon after this I was informed that he had taken the measles, from which it was believed he had recovered, though his general health was left impaired, and in which state it continued, gradually becoming worse, until the 11th of July, on which day he died.

*September* 1822.

